# Investigating Hydrogen in Zirconium Alloys by Means of Neutron Imaging

**DOI:** 10.3390/ma17040781

**Published:** 2024-02-06

**Authors:** Sarah Weick, Mirco Grosse

**Affiliations:** Institute for Applied Materials-Applied Materials Physics, Karlsruhe Institute of Technology, 76021 Karlsruhe, Germany

**Keywords:** neutrons, imaging, neutron imaging, hydrogen, claddings, zirconium, diffusion, in situ, radiography, tomography, nuclear

## Abstract

Neutrons interact with the magnetic moment of the atomic shell of an atom, as is common for X-rays, but mainly they interact directly with the nucleus. Therefore, the atomic number and the related number of electrons does not play a role in the strength of an interaction. Instead, hydrogen that is nearly invisible for X-rays has a higher attenuation for neutrons than most of the metals, e.g., zirconium, and thus would be visible through dark contrast in neutron images. Consequently, neutron imaging is a precise, non-destructive method to quantify the amount of hydrogen in materials with low attenuation. Because nuclear fuel cladding tubes of light water reactors are made of zirconium (98%), the hydrogen amount and distribution in metallic claddings can be investigated. Even hydrogen concentrations smaller than 10 wt.ppm can be determined locally with a spatial resolution of less than 10 μm (with a high-resolution neutron microscope). All in all, neutron imaging is a very fast and precise method for several applications. This article explains the basics of neutron imaging and provides samples of investigation possibilities, e.g., for hydrogen in zirconium alloy cladding tubes or in situ investigations of hydrogen diffusion in metals.

## 1. Introduction

Neutron imaging is a powerful, non-destructive method to investigate hydrogen in metals. Due to the high neutron cross section of hydrogen, it is easily detectable in materials with lower neutron cross sections, e.g., zirconium or steel. Several groups worldwide use neutron imaging for the investigation of hydrogen in nuclear fuel claddings made of zirconium alloys [1,2,3,4,5,6,7,8,9,10,11,12,13,14,15]. Because hydrogen usually affects the mechanical properties and, thus, the integrity of components used for many applications, e.g., nuclear fuel cladding tubes or pressure tubes in nuclear reactors, it is of high interest to detect hydrogen concentrations in materials locally, especially because hydrogen, as the smallest atom, diffuses very easily depending on gradients in concentration, temperature, chemical composition, and strain. In addition to concentration gradients, hydrogen diffusion, which strongly depends on hydrogen solubility, is initiated and/or accelerated with temperature gradients and stress gradients. With a calibration of the dependence of the neutron attenuation on the hydrogen concentration, neutron radiographs offer precise, quantitative determinations with spatial resolutions down to 50 μm in the usual measurements and to a few microns in neutron microscopy investigations. This depends on several factors, e.g., the pixel size of the detector system, the distance between the sample and the detector, the collimation, and the illumination time. With the high-resolution microscope of the Paul Scherrer Institute (PSI), even a spatial resolution of about 3 μm can be obtained. Regarding the hydrogen concentration, the detection limit of the hydrogen concentration is less than 10 wt.ppm [13]. It has to be mentioned that the resolution is multidimensional. The dimensions are the spatial resolutions in the three space directions; the contrast resolution is important for the resolution of the concentration determination; and the time resolution (important for in-situ measurements or for the number of samples that can be investigated in a given time) and neutron wavelength resolution are also involved. As shown in [16], the resolution dimension cannot be improved independently of the other ones. Improving one resolution dimension requires a deterioration of at least one other dimension.

The terminology “neutron imaging” covers not only neutron radiography, but also neutron tomography, where a quantitative 3D reconstruction of the hydrogen concentration within the sample can be made. Nevertheless, a tomography takes much more time (two or three orders of magnitude) than a single radiography, which has a standard illumination time between a couple of seconds and some minutes for a single neutron radiography frame. An advantages is the non-destructive character that allows for further investigations and analytical descriptions of the same sample. Further, destructive methods can be mutually used to verify the neutron imaging results. Not only ex situ, but also in situ experiments can be performed in the framework of hydrogen investigations in materials due to the high neutron transmission of several materials, for instance, those used for furnaces. Thus, samples can be monitored during their hydrogenation process and the following hydrogen diffusion and possible gradient-driven hydrogen movements within the sample. In the case of hydrogen in cladding tubes, this allows for in situ determination of the hydride reorientation process in cladding tubes due to stress-induced dissolution and recrystallisation processes. To give more examples besides application possibilities for engineering, biological investigations are feasible, because water or water-containing components, e.g., roots, have a very high neutron cross section, similarly to hydrogen, and thus can be investigated non-destructively and precisely by means of neutron imaging. This means that plants with roots and leaves, porous or water-rich rocks like limestone, fuel cells, engine oils, and more can be easily investigated. This paper will present more details regarding the investigation of hydrogen in cladding tubes that are used in the nuclear industry.

After explaining the basics of neutron imaging, this paper gives an overview about high-performance neutron imaging facilities in Europe. Further, examples related to investigations of the hydrogen behavior in zirconium alloys by means of neutron imaging are given.

## 2. Theory

Neutrons are electrically neutral, but have a magnetic moment. Thus, they interact with the magnetic moment of the electron shells of atoms, but mainly with the nucleus itself. The probability of an interaction between a nucleus and a neutron is described by the microscopic cross section *σ*. The interaction itself may be either an absorption of the neutron by the nuclei, an incoherent scattering process on the nuclei, or a coherent scattering process on the material’s structure. All interactions together can be referred to as the total neutron cross section *σ_tot_*, which can be summed up with the absorption cross section *σ_a_*, the incoherent cross section *σ_i_*, and the coherent scattering cross section *σ_c_*:

(1) $$\sigma_{tot}=\sigma_{a}+\sigma_{i}+\sigma_{c}$$

Examples of microscopic neutron cross sections relevant for the investigation of hydrogen in zirconium are presented in Table 1 for a neutron wavelength of 0.1798 nm.

The coherent scattering is caused by the average value of the scattering lengths of the lattice points. It results in constructive scattering effects with characteristic scattering patterns like small angle scattering, Bragg scattering, and Fresnel or Frauenhofer scattering. In contrast to coherent scattering, incoherent scattering does not produce characteristic patterns. The scattering intensity is equal for all directions and produces a constant background. This scattering is caused by point-to-point variations in the scattering lengths. In the case of hydrogen, the point-to-point variation in the proton spin interacting with the neutron spin results in a large variation in the scattering lengths and, thus, in a large, incoherent cross section. Another reason for incoherent scattering can be high concentrations of different isotopes of an element with differing neutron scattering lengths. In mixed crystals where one element substitutes the other, or in materials with high vacancy concentrations, the scattering length varies from point to point as well. This type of incoherent scattering is also known as monotonic Laue scattering [18].

The total macroscopic cross section, on the other hand, refers to the sum of the individual products of the number density *N* of the isotope *i* and the corresponding total microscopic neutron cross section $\sigma_{total_{i}}$:

(2) $$\Sigma_{total}=\sum_{i}N_{i}\;\sigma_{total_{i}}$$

The neutron’s wavelength, its kinetic energy, and the material’s structure are decisive factors for the probability of neutron absorption. Neglecting resonant absorption processes, the probability of such a process is higher if the wavelength is also higher. The neutron spectrum depends on the kind of source, for example, whether, e.g., thermal or cold neutrons are produced. The wavelength maximum of thermalized neutrons is in the range of 0.18 nm (25 meV), whereas the wavelength of cold neutrons thermalized in liquid-hydrogen vessels is in the range from 0.3 to <1.2 nm (9 meV to <0.6 meV).

**Figure 1:**
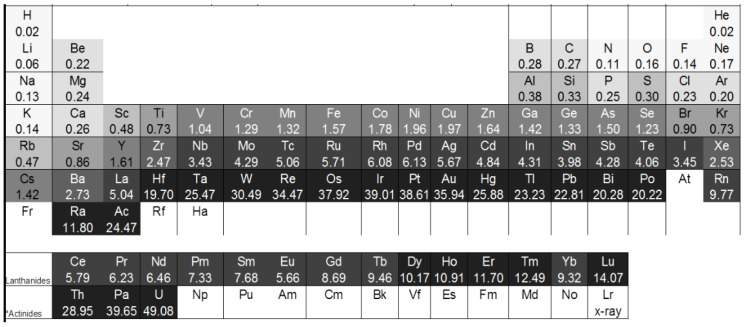
Periodic table with X-ray attenuation coefficients for 125 kV X-ray energies [19].

**Figure 2:**
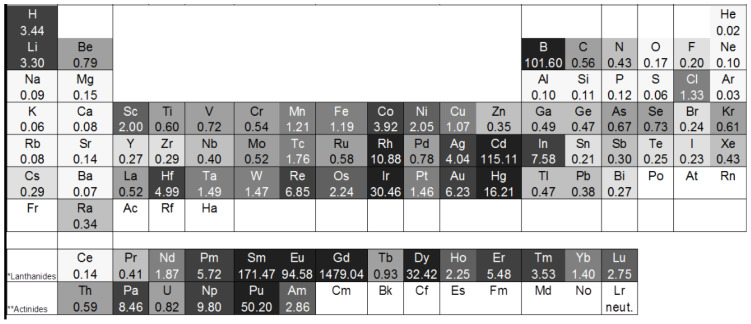
Periodic table with thermal neutron energy attenuation coefficients [19].

Contrarily to neutrons, the attenuation for X-rays depends on the atomic number of an element, because it increases with an increasing number of electrons in the shell. Thus, the attenuation coefficients for heavy metals are lower for neutrons than for X-rays, apart from cadmium and gadolinium. Elements with a high attenuation for neutrons that are appropriate for neutron imaging investigations are, e.g., hydrogen, lithium, and boron. These and further attenuation coefficients can be found in Figure 1 and Figure 2:

In order to calculate the attenuation, the intensities of the neutron beam must be measured before (*I*_0_) and after it passes through a sample. With additional information about the neutron path length through the sample *s* and Σ*_total_*, the attenuated intensity *I* is defined as:

(3) $$I=I_{0}\;\exp\left(-\Sigma_{total}\;s\right)$$

Additionally, a background noise *I_B_* must be considered for the determination of the correct Σ*_total_*. It should include all relevant disturbances during the measurements, e.g., electronic noise and any activated components or gamma radiations by the neutron source. All in all, the Σ*_total_* can be calculated as followed:

(4) $$\Sigma_{total}^{sample}=\frac{-\ln\left(\frac{I\;-\;I_{B}}{I_{0}\;-\;I_{B}}\right)}{s}$$

*I_B_* is the background intensity caused, for instance, by electronic noise or γ rays from activated components.

**Figure 3:**
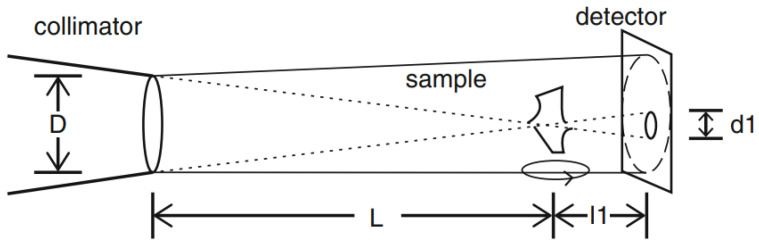
Schematic illustration of the projection of a sample’s shadow on the detector with the important *L* and *D* values that determine the quality of the neutron images [20]. A high-quality measurement should at least contain an *L*/*D* = 300.

Figure 3 illustrates the importance of beam geometry and a pinhole camera for neutron imaging applications. The aperture for a neutron beamline is much larger than for an X-ray tube. In order to make a compromise between a good spatial resolution and a good time resolution, and, thus, a sharp, good-quality neutron image in the end, it is important to choose appropriate *L* and *D* values for the measurements. With a larger *L* and smaller *D* and l1 values, the quality of the neutron image increases; thus, the aperture should be kept as far away and as small as possible. On the other hand, this leads to an intensity loss, which is why the sample should be placed right in front of or as close as possible to the detector. As can be seen in Figure 3, the neutrons follow the distance *L* from the aperture with the diameter *D* and then project a shadow of the sample on the detector. Thereby, their flight paths are not parallel.

## 3. Neutron Tomography

Instead of only investigating samples from a two-dimensional (2D) perspective, it is possible to reconstruct a three-dimensional (3D) tomography from several sample projections at different angles. For this purpose, the sample has to be rotated [21]. According to the sampling theorem for tomography investigations, the following number of projections *N_projections_* is needed:

(5) $$N_{projections}\;=\;\frac{\pi }{2}\;\frac{R_{sample}}{s_{pixel}}$$

where *R_sample_* is the radius of the circle including the whole sample and *s_pixel_* is the pixel size. For a precise reconstruction of tomography measurements with the usual pixel sizes, an amount of at least 400 images is crucial. Generally, the needed number of images can be calculated for each case using the maximum width (in pixels) of the sample projected on the detector.

**Figure 4:**
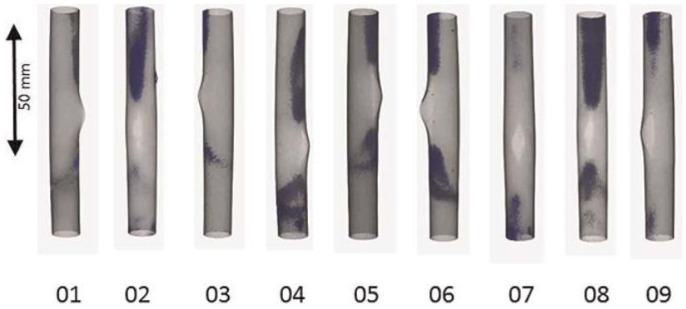
Three-dimensional reconstruction of the hydrogen distribution after a simulated design basis loss-of-coolant accident [17].

An example of a 3D reconstruction of hydrogen enrichments of fuel rod simulators from the QUENCH-LOCA-03 experiment is given in Figure 4. There, the hydrogen enrichments are visible through the dark contrast compared to the rod matrix.

## 4. Facilities

There are several high-performance neutron imaging facilities in Europe (Table 2):

### 4.1. NEXT at ILL (High Flux Reactor) in Grenoble

The neutron and X-ray imaging facility NEXT at the ILL in Grenoble (France) [22] has been in operation since 2016. The facility uses cold neutrons and offers a spatial resolution of up to 5 µm. The maximal field of view is 170 mm × 170 mm at a spatial resolution of 83 µm. The high-flux ILL research reactor provides cold neutrons with a flux of 3 × 10^8^ n/cm^2^/s at a collimation of *L*/*D* = 333.

### 4.2. ANTARES at FRM-2 in Garching

The neutron imaging facility ANTARES at the FRM-2 reactor in Garching (Germany) [23] will be restarted in the second half of 2024. The neutron source provides a cold neutron flux of 6.4 × 10^7^ n/cm^2^/s at a collimation of *L*/*D* = 500. Roughly estimated, it has about half of the flux of NEXT.

### 4.3. Neutron Imaging Instruments at SINQ, PSI in Villigen

**Figure 5:**
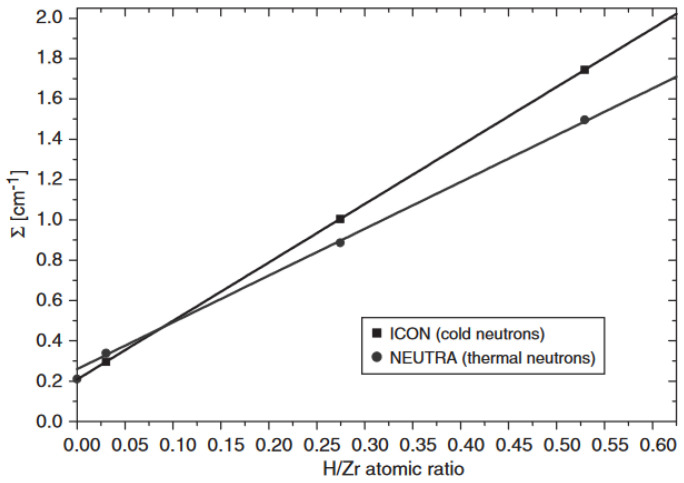
Comparison of the total cross sections of the thermal NEUTRA and the cold ICON spectra [4].

At the Swiss spallation neutron source SINQ, several beamlines are used for neutron imaging. Dedicated to neutron imaging are the NEUTRA and ICON facilities. The NEUTRA facility [24] uses thermal neutrons with flux of about 6 × 10^6^ n/cm^2^/s for *L*/*D* = 350 and a usual proton current on the SINQ target, producing neutrons of 1.2 mA. The ICON facility [25] has a cold spectrum neutron flux of 1.5 × 10^7^ n/cm^2^/s for *L*/*D* = 343 and a SINQ proton current of 1.2 mA. The difference in the hydrogen dependencies on the total macroscopic neutron cross sections between the spectra of the two facilities is given in Figure 5. It is visible that the total macroscopic neutron cross section for hydrogen-free Zircaloy-4 is higher for thermal neutrons, but the effect of hydrogen is lower for the thermal spectrum than for the cold one. In addition to the NEUTRA and ICON beamlines, the beamlines BOA and POLDI are available, at least partially, for imaging experiments. The POLDI facility is a neutron diffractometer dedicated to strain measurements [26]. Currently, it is partially used for neutron microscopy [27]. The thermal neutron spectrum is comparable to that of the NEUTRA facility. The BOA beamline [28] was originally dedicated to the testing of neutron optical components. The neutron spectrum is even colder than that of ICON. The neutron flux of 2.7 × 10^7^ n/cm^2^/s for *L*/*D* = 400 is given for BOA.

### 4.4. IMAT at ISIS in the STFC Rutherford Appleton Laboratory at the Harwell Campus Didcot

The time-of-flight neutron imaging facility IMAT [29] offers a high wavelength resolution. The flux depends on the wavelength. The integral flux is 1.4 × 10^7^ n/cm^2^/s for *L*/*D* = 500, which is comparable to the flux provided at BOA.

In addition to these high-performance facilities, possibilities for neutron imaging exist in Swierg (Poland), Budapest (Hungary) and Dubna (Russia). Outside of Europe, high-performance neutron imaging facilities include RADEN at J-Parc Tokai (Japan) [30] and DINGO at ANSTO (Australia) [31]. In the USA, further facilities are available and in operation: NIF at NIST [32], ERNI at LANSCE [33], and MARS [34] and VENUS [35] at ORNL. New high-performance neutron imaging facilities are currently under construction in China and Sweden.

## 5. Examples for Neutron Experiments

### 5.1. Hydrogen Diffusion Studies

As already mentioned, hydrogen uptake is a critical degradation/corrosion phenomenon for metals in various applications. In the framework of nuclear reactors, hydrogen can be taken up by the metallic cladding that surrounds nuclear fuel pellets or pressure tubes in CANDU reactors. Because these tubes consist mainly of zirconium and are in direct contact to the surrounding cooling water during operation, the following corrosion reaction takes place:

Zr + 2H_2_O = ZrO_2_ + 4H (6)

Because of the solute state of the hydrogen during operation, the integrity of the cladding tubes and, thus, the fuel rods remain intact. However, several phenomena come along with absorbed hydrogen and are relevant for safety.

### 5.2. Analysis of the Hydrogen Distribution in Cladding Tubes after Simulated Basis Loss of Coolant Accidents

**Figure 6:**
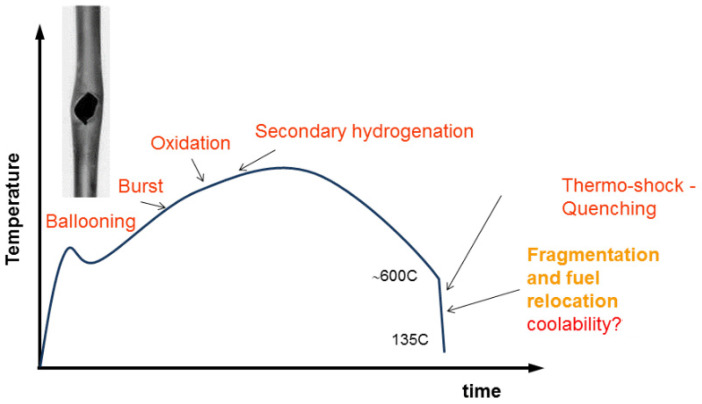
Scheme of a LOCA scenario dependent on temperature and time [38].

The so-called secondary hydrogenation during the design basis loss of coolant accident (LOCA) scenarios (see Figure 6) degrade the toughness of the cladding tubes. This occurs after bursting of the tubes and an ingress of steam into the claddings. Then, highly hydrogen-rich zones can form, which increase the risk of brittle fractures due to thermal shock during the emergency cooling. Consequently, fuel pellets can be relocated, fission products can be released, and the coolability of the reactor core cannot be ensured any longer. Many studies focus on investigations of LOCA scenarios [36] with newly developed cladding materials and/or coatings [37].

Eight large-scale tests simulating LOCA were performed at KIT. The tests were performed using a bundle of 21 fuel rod simulators, with cladding tubes made of zirconium alloys used in Germany (Zircaloy-4 (Framatome, Germany), ZIRLO™ (Westinghouse, Sweden), and M5^®^ (Framatome, Germany)). The fuel rod simulator bundles had a prototypical geometry. The test protocol was developed according to the German nuclear rules for design-basis, large-break LOCA: heating in steam up to about 1000 °C at a rate of 5 to 6 K/s; cooling down for three minutes to about 600 °C, and water quenching to terminate the test. Neutron imaging was a very important part of the post-test examinations. Neutron radiography was performed with all cladding tubes, whereas tomography measurements were applied only at chosen ones, as they had had shown clear hydrogen enrichment in the neutron radiographies. The facilities ICON and BOA at PSI, the ANTARES beamline at TU Munich, and the CONRAD [39] facility at the Helmholtz Center Berlin (now out of operation) were used for the neutron imaging investigations. Pixel sizes of about 30 to 40 µm were applied. More details are given by Stuckert et al. [36]. As an example, Figure 4 shows the results of the hydrogen distribution in nine claddings of the QUENCH-L3 test using ZIRLO™ tubes. The hydrogen-enriched positions are darker. Two types of hydrogen-enriched locations can be divided: firstly, hydrogen-enriched rings (c.f. Figure 4), which are non-perpendicularly oriented to the tube axis; and secondly, hydrogen-enriched strips parallel to the tube axis. The tomography results were analyzed statistically by determining the maximum mean and minimum CT values for each axial position inside the tube wall. Using the calibration, the hydrogen concentrations can be derived. Hydrogen concentrations up to 1500 wt.ppm were detected.

### 5.3. Hydrogen Diffusion in Zirconium Alloys

A possible reorientation of zirconium hydrides in the cladding tubes after the vacuum-drying process of spent nuclear fuel (SNF), where temperatures of up to 400 °C are reached, can affect the integrity of cladding tubes. Such a reorientation is mainly caused by

- The fabrication history;
- The material’s texture;
- The stress state in the cladding tube connected to the higher hoop stress generated by the fission products of the pellets.

**Figure 7:**
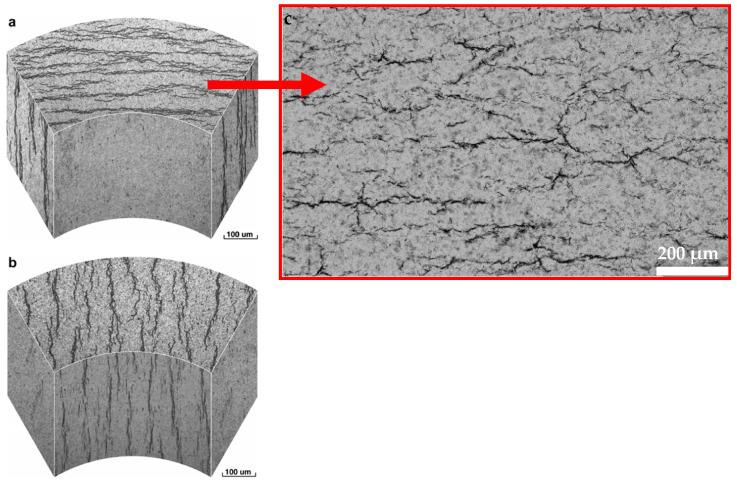
Illustration of circumferentially (**a**) and radially (**b**) oriented zirconium hydrides (black) in a cladding tube [40] with a light microscopic image of a Zry-4 cladding tube sample with mainly circumferentially orientated zirconium hydrides (**c**).

This will lead to hydride precipitation in the radial direction of the cladding tube. This precipitation in the radial direction may occur after a dissolution of already-precipitated hydrides in the circumferential direction, triggered by a temperature-controlled accessing of the terminal solubility limit for dissolution (TSSd). This solubility limit is further controlled by the hydrogen concentration within the sample and other parameters. Investigations based on reorientation processes of zirconium hydrides in cladding tubes are very sought after [40,41,42]. An illustration of hydrides oriented in the circumferential and radial directions is given in Figure 7a,b, in addition to a micrograph of a Zircaloy-4 (Zry-4) cladding tube sample with mainly circumferentially and a few radially orientated zirconium hydrides. The sample was hydrogenated at 900 °C in pure hydrogen gas in a Sievert’s chamber until a concentration of 230 wt.ppm hydrogen had been taken up by the cladding and homogeneously diffused (Figure 7c).

**Figure 8:**
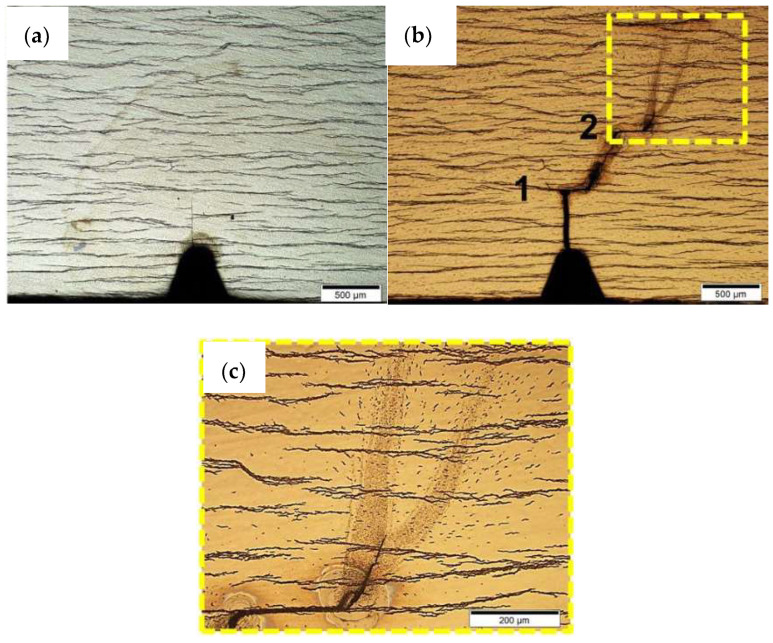
Example of DHC development in a Zr-2.5%Nb sample before (**a**) and after (**b**,**c**) an applied load at 250 °C [15].

The delayed hydride cracking (DHC) phenomena illustrated in Figure 8 can occur during operation or during the dry storage of SNF. The cladding tube fails after hours of constant mechanical load without any creeping. Hydrogen diffuses to a crack tip and precipitates there until a certain length of a hydride is reached. Then, breaking and further crack propagation are caused, and the whole process is repeated until the tube fails. The driving force of this process is the stress gradient created by the plastic deformed zone ahead of the crack tip in the bulk material. Neutron imaging serves as a non-destructive method by which to analyze the hydrogen behavior during the DHC process, and should be used for future studies to experimentally investigate the phenomenon in situ. Nevertheless, Soria et al. [15] have already been able to perform similar tests at 250 °C with two different zirconium alloys. Some of the results are shown in Figure 8, where the pathway of the crack propagation in one sample is shown during the in-situ experiment.

**Figure 9:**
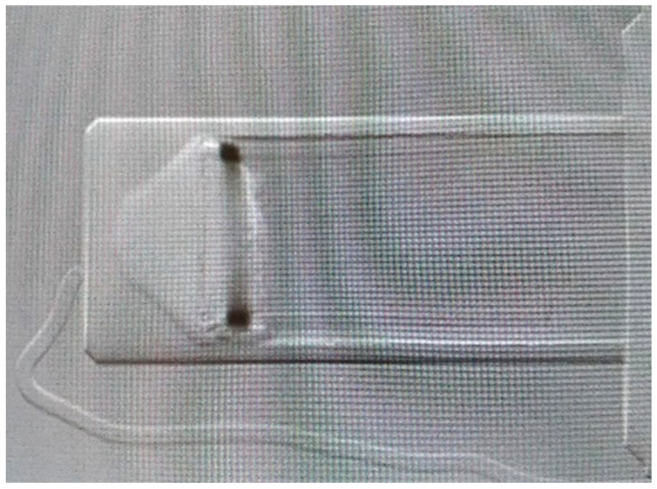
Neutron radiograph of an in situ hydrogen diffusion experiment with ZrH_2_ powder in a Zry-4 cladding tube at the BOA beamline at the PSI.

Nevertheless, precise determinations of the hydrogen diffusion process, especially in cladding tubes, are only available to a limited extent. The reasons are the lack of experiments under real scenario conditions and the limited consideration of influencing parameters like the stress state, the texture, or the grain size. Nevertheless, hydrogen in cladding tubes/zirconium can be easily investigated using destructive methods such as metallography or carrier hot gas extraction (CHGE). But non-destructive investigations, and, thus, neutron imaging, offer more possibilities, even to observe samples in situ during different processes, e.g., crack initiation, crack development, ruptures, or diffusion (Figure 9) at high temperatures. Concerning diffusion experiments, the relevance of diffusion coefficients and, thus, the diffusion velocity dependent on its direction in the material’s structure are of importance. The method of determining diffusion coefficients from neutron radiography data is described in Equation (7). If the diffusion coefficients are modeled [43] instead of experimentally determined, most, but not all, relevant parameters are taken into account; thus, the literature values obtained from pure zirconium with undefined textures and grain sizes are not comparable to the cladding tube materials. Hydrogen uptake starts when the very thin oxide layer which was formed at room temperature in air is dissoluted. The time needed for this dissolution is unknown because it depends on many parameters, like the humidity of the laboratory air, the time between polishing the sample and exposing it in the furnace, the quality of the vacuum, and the inert atmosphere in the furnace. As a consequence, the starting time of the diffusion process is difficult to specify. This is one intention of in situ neutron imaging and the experiment presented in Figure 9. Here, the beginning of the hydrogen uptake is clearly visible by changing the neutron transmission at the position of the hydrogen absorption. It is planned to determine the hydrogen diffusion coefficients for all relevant macroscopical axis directions, because they differ in their c- or a-axes. For the experiment, a Zry-4 cladding tube was peroxidized in air at 450 °C for several hours to produce an oxide layer a few micrometers thick that would prevent hydrogen uptake. A feasibility test was performed with a leak-tight sample capsule at the BOA facility at PSI, while using a capsule filled with the sample on ZrH_2_ powder in a helium atmosphere. Hydrogen was taken up by the sample at the intended/grinded position. However, the large distance between the sample inside the furnace and the detector strongly reduced the spatial resolution, because a very good collimation of the beam of *L*/*D* > 1000 was not available with sufficient intensity at this beamline. Additionally, the furnace which was used needed 3 h to reach the predefined temperature. During the heating-up process, diffusion occurred. The diffusion coefficients were not determined precisely enough. Subsequent investigations are still ongoing. The results will be published elsewhere.

**Figure 10:**
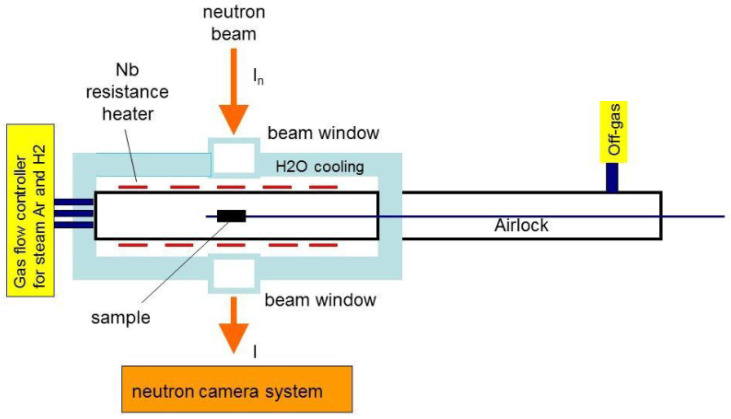
Scheme of the portable INRRO furnace of the Karlsruhe Institute of Technology [5].

Different materials/metals have different diffusion coefficients depending on the crystallographic direction, e.g., hydrogen in zirconium depending on the diffusion direction in a hexagonal crystal lattice. Neutron radiography experiments, as performed by, e.g., Beyer et al. [44,45], shall help to determine precise values for this process. In the case of cladding tubes that consist mainly of zirconium, an additional difficulty is that the material immediately forms a zirconium oxide layer when it is in contact with air. Therefore, methods to investigate the hydrogen uptake process in situ after the dissolution of the oxide layer are suitable. In this context, measurements with the so-called in situ neutron radiography reaction (INNRO) furnace (Figure 10) at the ICON beamline at the PSI were performed.

Because of the large distance of about 300 mm between the sample inside the furnace and the detector, a collimation of *L*/*D* = 800 was applied, which decreased the neutron flux at the sample. In order to obtain a good contrast resolution for a precise determination of the local hydrogen concentration, each radiograph was illuminated for 120 s. The samples were solid cylinders 20 mm in length and 12 mm in diameter, and were made of Zircaloy-4. The samples were pre-oxidized at 1100 °C for 1 h to produce a 50 µm oxide layer on the surface, preventing hydrogen uptake.

**Figure 11:**
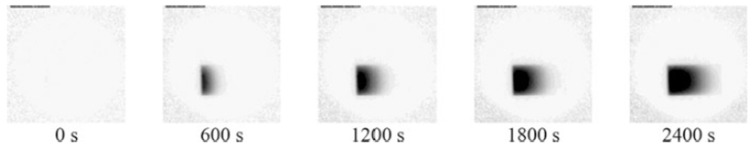
In situ neutron radiography image sequences of the hydrogen diffusion in a Zry-4 cladding tube at 900 °C, taken at the ICON beamline of the PSI [46].

The sample was heated at a rate of 10 K/min to the test temperature in flowing argon (flux: 50 L/h). After reaching the test temperature, hydrogen was injected with a flux of 4 L/h, in addition to the argon flux hydrogen. The isothermal tests were performed at 900, 1000, 1100, 1200, and 1300 °C. Figure 11 shows neutron radiographs taken at different diffusion times at 900 °C. The images were compared to the image of the initial state. Therefore, only changes in the neutron transmission became visible. The diffusion of the hydrogen (dark) could be observed visually during the measurements, without processing the data. From each neutron radiograph, the hydrogen distribution can be determined via the gray value distribution.

The hydrogen concentration can be fitted using the analytical diffusion equation by assuming a one-sided infinite sample—*c* is the hydrogen concentration; *c*_0_ is the hydrogen concentration in equilibrium to the hydrogen partial pressure in the surrounding gas atmosphere at the test temperature, according to Sieverts’ law; *x* is the distance to the base plane through which hydrogen was absorbed; *D* is the diffusion coefficient; and *t* the time:

(7) $$c\left(x,t\right)=c_{0}\left(1-\mathrm{erf}\left(\frac{x}{2\sqrt{Dt}}\right)\right)$$

In order to determine the exact quantitative hydrogen content in the samples, it is advantageous to measure several calibration samples with different and known hydrogen contents under the same conditions. These calibration samples were produced by annealing the same material in hydrogen containing an inert gas until equilibrium between the hydrogen concentration in the sample and the hydrogen partial pressure in the gas atmosphere was reached. The hydrogen concentration in the sample was determined by a measurement of the weight gain of the sample. Sieverts’ law describes the equilibrium correlation between the ratio of the number densities of hydrogen *N_H_* and zirconium *N_Zr_* and the hydrogen partial pressure in the gas atmosphere *p*_*H*2_:

(8) $$c_{H}^{metal}=\sqrt{k_{S}\;p_{H_{2}}}$$

with *k_S_* as the Sieverts’ coefficient depending on the Arrhenius type.

**Figure 12:**
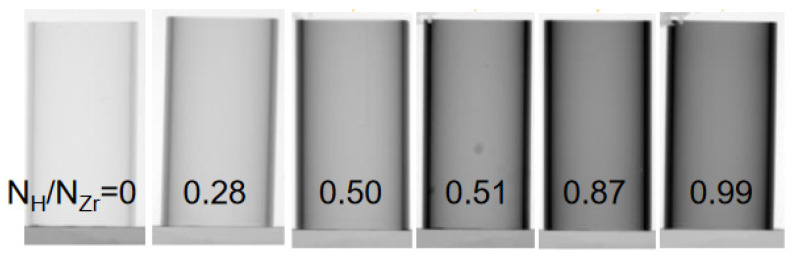
A calibration sample set of Zircaloy-4 (Zry-4) cladding tube samples with heights of 20 mm, where the neutron attenuation increases with an increasing amount of hydrogen in the metal. The neutron radiographs were taken at the ICON beamline at the PSI.

Figure 12 compares neutron radiographs of the calibration samples with different ratios of hydrogen and zirconium number densities.

**Figure 13:**
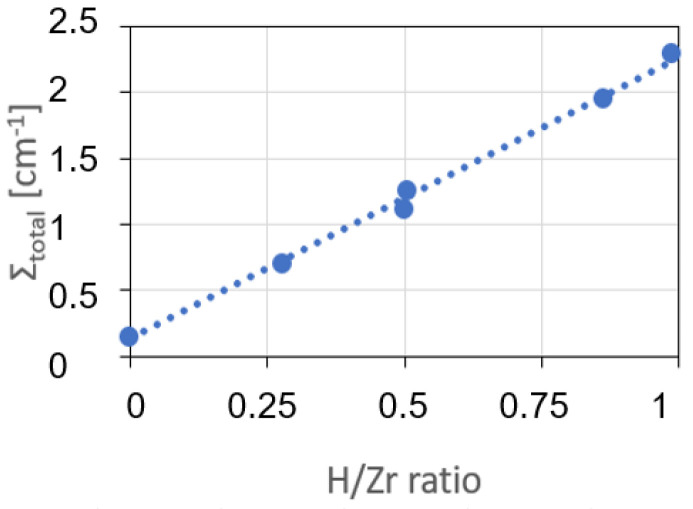
Calibration curve plotted as the relation between the total macroscopic neutron cross section $\sum_{total}$ and the number density ratio between hydrogen and zirconium *N_H_*/*N_Zr_*. The measurements were made at the ICON beamline at the PSI.

With the intensity measurements of the calibration samples, a calibration curve of the correlation between the total macroscopic neutron cross section and the number density ratio, according to Equation (2), can be determined as shown in Figure 13. With its slope, it becomes possible to calculate the previously unknown hydrogen concentrations of the measured samples. It is essential to measure the calibration samples during each beam time, even if the same beamline is used. Only then can the effects of setup changes or neutron spectrum changes be excluded. These effects are well described by [30].

It must be mentioned that the calibration curve determined for the tube-shaped samples from 3D analyses of neutron tomography data differs from the ones measured by means of neutron radiography (Figure 13). The differences increase with increasing hydrogen concentrations. The calibration curves are no longer linear, but flattened at the higher-concentration side of the diagram. The reason is that 3D reconstruction also uses positions with longer neutron path lengths. This can be associated with hardening of the neutron spectra and a stronger influence of multiple scattering. Therefore, if the CT value is analyzed quantitatively, tomography measurements of the calibration samples are mandatory.

## 6. Conclusions

Neutron imaging methods are sufficient for the investigations concerning hydrogen-related processes in nuclear cladding and pressure tubes made of zirconium alloys. The high total neutron cross section of hydrogen and the low one of zirconium allows for the detection of even small amounts of hydrogen in zirconium-based alloys. It enables the fast determination of hydrogen, with a spatial resolution of tens of microns with standard radiography or tomography measurements, and even up to few microns with neutron microscopy experiments.

The high neutron transmission of various materials offers the possibility of in situ experiments in well-defined sample environments, such as, for instance, in furnaces.

The correlation between the total macroscopic neutron cross section and the hydrogen concentration can be determined quantitatively using samples with well-known homogeneous hydrogen concentrations. A resolution of only a few wt.ppm is possible.

New methodical developments on the field of neutron imaging will provide promising possibilities for material investigations in the future. For instance, neutron microscopy offers spatial resolutions of less than 5 µm. Imaging experiments with short, pulsed beams allow the measurements of the lateral distribution of the Bragg edges to determine local stresses, textures, or the separation of special objects with given structures from complex micrographs.

However, a problem is the strong overbooking of the neutron imaging facilities, which will be difficult to solve. Therefore, the use of more beamlines for imaging experiments would be helpful.

## Figures and Tables

**Table 1:** Microscopic neutron cross sections for hydrogen, oxygen, and zirconium [17].

Element	*σ_a_* (10^−24^ m^2^)	*σ_c_* (10^−24^ m^2^)	*σ_i_* (10^−24^ m^2^)	*σ_total_* (10^−24^ m^2^)
H	0.333	1.757	80.26	82.02
O	0.00019	4.232	0.0008	4.233
Zr	0.185	6.44	0.02	6.645

**Table 2:** Comparison of high-performance neutron imaging facilities in Europe.

Beamline	Place	Flux (n/cm^2^/s)	*L*/*D*
NEXT	Grenoble, FR	3 × 10^8^	333
ANTARES	Garching, DE	6.4 × 10^7^	500
NEUTRA	Villigen, CH	6 × 10^6^	350
ICON	Villigen, CH	1.5 × 10^7^	343
BOA	Villigen, CH	2.7 × 10^7^	400
IMAT	Oxfordshire, UK	1.4 × 10^7^	500

## Data Availability

Data are contained within the article.
